# Parents Support Implementation of HIV Testing and Counseling at School: Cross-Sectional Study with Parents of Adolescent Attending High School in Gauteng and North West Provinces, South Africa

**DOI:** 10.1155/2016/4842814

**Published:** 2016-10-11

**Authors:** Sphiwe Madiba, Mathildah Mokgatle

**Affiliations:** ^1^School of Public Health, Department of Environmental and Occupational Heath, Sefako Makgatho Health Sciences University, Pretoria, South Africa; ^2^School of Public Health, Department of Biostatistics, Sefako Makgatho Health Sciences University, Pretoria, South Africa

## Abstract

*Background*. A formative assessment of the implementation of HIV testing and counseling (HTC) at school showed high acceptability and willingness to test among learners. However, the success of the proposed HTC depends on the support and acceptability of key stakeholders, including the parents. The aim of the study was to assess the opinions and acceptability of the implementation of HTC at school among parents of adolescents in high school.* Methods*. This was a cross-sectional household survey conducted with parents of adolescents attending high schools in educational districts in North West and Gauteng provinces, South Africa.* Results*. A total of 804 parents participated, and 548 (68.3%) were biological mothers, 85 (10.6%) were fathers, and the remaining were other relatives including grandmothers. Almost all (*n* = 742, 92.9%) parents were in support of implementation and provision of HTC at school, 701 (87.7%) would allow their children to be tested at school, 365 (46%) felt that parental consent was not needed to test at school, and 39.4% preferred to receive the HIV test results with their children.* Conclusion*. Parents accept the roll-out of an HTC program at school and have a role to play in supporting children who test positive for HIV.

## 1. Introduction

A formative evaluation was conducted in 2013 to assess the opinions of high school learners about the implementation of HIV testing and counseling (HTC) in schools and showed high acceptability of HTC and willingness among learners to test at school [1]. When the South African National Department of Health proposed to implement HTC at schools in 2011 as part of the national HIV testing and counseling (HTC) campaign which aimed to target 12-year-old to 60-year-old individuals [2], there were concerns from key stakeholders about the appropriateness of HIV testing at schools. Although the majority (84%) of learners were willing to test at school [1], the success of the proposed roll-out of HTC at school depends on the support and acceptability of key stakeholders, such as educators, school governing bodies, parents/guardians, and healthcare providers.

In May 2015, the Department of Basic Education released the Draft National Policy on HIV, STIs, and TB to guide comprehensive responses to HIV and TB, with prevention being one of the key themes of the policy. The policy proposes to provide, via mobile health units, counseling on sexual and reproductive health issues to all senior, further education and training (FET) phase, and intermediate learners, where required. This should include the provision of dual protection contraception, HTC, and screening for STIs. Under roles and responsibilities, the policy proposes that parents and communities will be required to participate in the school response to HIV and TB and the implementation of programs at the school level. Their support, resources, and capacity will be harnessed to play a supporting role and will be enhanced through guidance and training [3].

The need to involve parents as part of the comprehensive strategy for improving adolescents' health and development and prevention of HIV/AIDS has been advocated in previous research [4, 5]. This advocacy relies on the assumption that parents are knowledgeable enough to teach their children about sexual topics including HIV/AIDS. However, there is no evidence to assume that parents are knowledgeable enough and fully equipped to teach their children about HIV/AIDS [4]. This lack of evidence shows that parents are an understudied and underutilized resource in HIV/AIDS interventions, particularly in sub-Saharan Africa [6].

As already mentioned, parents were identified as one of the key stakeholders in the implementation of the proposed HTC at school. There has not been any empirical data on the role of parents in school based HIV testing in South Africa and other sub-Saharan countries. HTC services in most countries in sub-Saharan Africa are offered in clinics, in hospitals, through a door-to-door approach, and through outreach campaigns. There are no government-driven school based HTC services as yet [1]. The provision of HTC at school is still a proposal by National Departments of Health and Basic Education [3], and there is very little documentation on the provision of HTC at school [1, 7, 8]. There are two documented school based HTC programs in the literature: one is in South Africa, where a nonprofit organisation in northern KwaZulu-Natal offered HTC at school using drama to encourage learners to test [8], and the other is in Uganda, where a mission hospital used a mobile service to provide HTC services in school settings [9].

The formative study conducted by the investigators to assess the acceptability of HTC at school [1] was a first in South Africa. As already stated, HTC at school is not yet implemented and there are no empirical data to inform the implementation of the proposed HTC at school. Since parents were identified as key stakeholders in the implementation of the proposed HTC at school, understanding their views and attitudes towards the provision of HTC for learners at school will inform the procedures and strategies for the provision of HTC in schools. The present study was conducted to assess parents' opinions and acceptability of the proposed HTC at school and determine their perceived role in the implementation of HTC. The involvement of parents in the implementation of HTC at school will require open communication about HIV and other sexual matters between parents and adolescents in order for parents to support the adolescents in the event that they test positive for HIV. The study further determined parental perceptions on parent-child communication about sexual and reproductive health.

One of the arguments regarding the appropriateness of HTC at school is that learners who test positive might be discriminated against. Research has shown that negative attitudes towards HIV-positive people are barriers against the uptake of HIV prevention strategies [10, 11]. In the case of the proposed roll-out of HTC at school, it is believed that parental negative attitudes towards learners who test positive will influence acceptability of HTC at school. We therefore examined parental attitudes towards learners who test positive for HIV and their interaction with other children at school. The study findings will inform the development of appropriate interventions to empower parents with adequate skills to initiate or improve discussions about sexual matters with adolescents [12] in their supportive role in the implementation of HTC at school.

## 2. Methods

### 2.1. Study Design

This was a cross-sectional study conducted from April to August 2014 among parents or guardians of children who were attending high schools. The current study is a follow-up of a formative assessment survey conducted to assess high school learners' acceptability of HTC at school. The original survey was conducted among 3978 students in grades 10–12, selected from 17 high schools in educational districts in North West and Gauteng provinces, South Africa [1].

### 2.2. Study Setting

The current study was conducted among parents of learners attending the high schools, where the formative assessment of HTC was conducted (17 high schools). Of the 17 high schools included in the sample, nine were selected from two rural subdistricts in North West province and eight were selected from an urban district in Gauteng province. The school setting was selected as most appropriate to access parents whose children attended the same schools, where the previous formative assessment was conducted. The research team consisting of a research coordinator and research assistants visited the selected schools to explain the purpose of the study and the plan to use the learners to deliver the questionnaires to their parents. The sampling procedure was similar to that adopted for the study conducted with learners; the Life Orientation (LO) teacher facilitated the sampling and recruitment process. In all the schools, the LO periods on the school time table for grades 10–12 were randomly assigned to the research team to distribute the questionnaire to the students who volunteered to transport the questionnaire to parents. As stated, the assigning of LO periods was random and the selected classes were not necessarily the same as those selected for the study with learners because the current study did not attempt to pair parents to children but aimed to recruit parents from the same schools where the previous formative assessment was conducted.

### 2.3. Data Collection

Students in the assigned LO classes were requested to deliver and return the survey package which consisted of an information leaflet, a structured questionnaire, and informed consent in a sealed envelope to one of their parents and then back to the school after the questionnaire had been completed and sealed in a second envelope (parent refers to any primary caregiver, including biological parents, grandparents, uncles, aunts, and older siblings). The information leaflet provided adequate instructions on how to complete the questionnaire and on the voluntary nature of the study. Parents were also informed that nonparticipation would not affect their child's learning negatively. They were also informed that if they agreed to participate, they should sign the consent form and complete the questionnaire and return it to the school within four days of receipt. Learners were informed that only one parent/guardian in the household should complete the survey. This instruction was necessary to ensure that if siblings from different classes received the survey package, they should inform their parent/s that only one questionnaire should be completed per household to avoid duplication, which would affect the reliability of the study findings. The LO teachers from the selected classes received the returned envelopes in a sealed box which were then collected by the research team. The LO teachers also reminded the students to return the questionnaires within the stipulated time of four days.

### 2.4. Measures

A structured, self-administered questionnaire was used to obtain data from parents who were asked to supply sociodemographic information such as age, sex, marital status, educational level, employment, income, and family setup. Parents also provided information on the age, sex, and grade and their relationship with the child who delivered the questionnaire. To answer the questions on whether parents communicated with their children about sexuality, parents were asked to indicate whether they had ever had a discussion about sexual issues with their children and whether parent-child communication about sexuality was important and the common barriers to communication about sex and related topics with adolescent children. To assess the parents' HIV testing practices, uptake of HTC was measured by asking whether they had ever been tested for HIV, and for those who indicated that they had, they were asked about the place the HIV testing took place, the last time they had an HIV test, and the reasons for testing. To assess acceptability of HTC at schools, parents were asked whether providing HTC at school was a good idea and whether they would allow their children to be tested at school, should HTC be implemented. Lastly, their attitudes towards HIV-positive learners were assessed using six questions in the form of agree/disagree/not sure. We used a validated questionnaire used by the investigators to assess learner's acceptability of HTC in school [1]. The questionnaire was in English as more than five official languages are prevalent in the study setting; the translation and distribution of the questionnaire to the parents according to the home language were going to make recruitment complex.

### 2.5. Ethical Approval

Ethical approval was obtained from the Research and Ethics Committee of the University of Limpopo, Medunsa Campus (MREC/H/45/2014: IR).

### 2.6. Data Analysis

Data analyses were performed with Stata version 13. Descriptive statistics were computed and frequency distributions were used to summarize sociodemographic data. To assess the attitudes towards HIV-positive learners, we assigned a score of 1 for every positive answer in the attitude towards HIV-positive learners section and 0 for negative answers. Attitude scores ranged from 0 to 6, and the mean score was determined, with scores below the mean being considered as a negative attitude, and scores equal to and more than the mean were considered as positive attitude. Univariate and multiple logistic regressions were performed to assess the association between the outcome variable (ever been tested for HIV, acceptability of HTC, and parental concern for HTC) and independent variables like parental sociodemographic variables. The adjusted and unadjusted odds ratios (OR) were calculated and confidence intervals (CI) were set at 95%, and *P* values of <0.05 were considered to be statistically significant.

## 3. Results

### 3.1. Sociodemographic Description of Parents

The sociodemographic characteristics of the parents are reported in Table 1. There were more female, 691 (86.3%), than male, 110 (13.7%), parents, and the median age was 43 years, with an IQR of 38–50 years. Over two-thirds (*n* = 548, 68.3%) of the parents were biological mothers, 301 (37.4%) had completed high school, and over half (*n* = 439, 54.5%) were unemployed, and the main source of income for almost all (*n* = 375, 92.4%) was the old age and child support grants. For those who were employed, slightly over a quarter (*n* = 92, 27.3%) earned less than R2000.00 South African Rand per month, while only 60 (17.8%) earned above R5000.00 South African Rand. In terms of family composition, the majority (*n* = 396, 49.5%) were single headed households.

The parents provided demographic data about the index adolescent (the learner who brought the survey home). There were more females (*n* = 576, 72.5%) than males (*n* = 219, 27.6%). The mean age of the adolescents was 17.1 years (SD = 1.49, range: 13–21 years); the majority (*n* = 531, 67.5%) were aged between 16 and 18 years, with only 114 (14.5%) aged 15 years and younger. In terms of the level of education, the majority (*n* = 317, 40.3%) were in the 10th grade, over a third (*n* = 262, 33.3%) were in the 11th grade, and over a quarter (*n* = 204, 26%) were in the 12th grade.

### 3.2. Parents' Sexuality Discussion with Children

Table 2 presents parents' responses on discussing sexuality with their adolescent children. The majority (*n* = 652, 81%) indicated that they had communicated with their children on sexual issues at some time, more than half (*n* = 440, 54.9%) initiated the conversations to prepare their adolescent children for sexuality, and almost half (*n* = 393, 49.3%) indicated that it was not easy to do so.

### 3.3. The HIV Testing Practices of Parents

Table 3 presents parents' HIV testing practices, and the majority (*n* = 703, 87.9%) indicated that they had been tested for HIV, about two-thirds (*n* = 420, 59.7%) accessed HTC in a clinic, over a third (*n* = 243, 34.6%) were tested in the past three months, majority (*n* = 594, 84.3%) tested to know their status, and 367 (46%) were of the opinion that it was not easy for adults to test for HIV. Of the 97 participants who had never been tested, almost half (*n* = 42, 43.8%) indicated that they never thought about going for an HIV test, and 22 (22.9%) did not believe that they could be infected with HIV. Logistic regression showed no association between the sociodemographic variables and ever tested for HIV.

### 3.4. Parents' Views about Implementation of HTC at School

Almost half (*n* = 389, 48.9%) of the parents felt that it was not easy for learners to test for HIV in the current HTC services. To assess the acceptability of HTC at schools, parents were asked to indicate if the provision of HTC for learners at schools was a good idea, the vast majority (*n* = 742, 92.9%) were in support of the provision of HTC at school, and 701 (87.7%) would let their children test at school should HTC be implemented. Regarding parental consent for HIV testing at school, almost half (*n* = 365, 46%) of the parents felt that parental consent was not necessary for learners to be tested at school. They also indicated their preferences as to how the HIV test results should be handled after testing at school, with slightly less than half (*n* = 375, 47.1%) indicating that the result should be given to the learner alone at school. Concerning disclosure of HIV status should a learner test positive, the majority (*n* = 545, 68.3%) felt that children should disclose the status to parents (Table 4).

Univariate analysis and logistic regression showed no association between acceptability of HTC and sociodemographic variables. However, logistic regression showed a significant association between need for parental consent and sociodemographic variables. Need for parental consent was associated with parental relationship with learner (OR = 0.90, *P* = 0.033, and CI: 0.82–0.99), family structure (OR = 0.90, *P* = 0.048, and CI: 0.81–0.99), and issuing of HTC results (OR = 1.77, *P* = 0.000, and CI: 1.44–2.177). Parents who preferred that the HTC results should be given to the child with the parent present were four times more likely to suggest parental consent for HTC (OR = 4.18, *P* = 0.000, and CI: 3.02–5.78) than those who preferred that they be given to the child alone.  Table 5 shows that preference for issuing HTC result was independently associated with the need for parental consent at multivariate analysis.

### 3.5. Attitudes towards HIV-Positive Learners

The attitudes of the parents towards learners who are HIV-positive were assessed using six questions, and the scores ranged from 0 to 6 (M = 4.75; SD = 0.92). The results indicate that the majority (*n* = 560, 69.6%) of the parents had positive attitudes towards HIV-infected learners; they scored more than the mean score of 4.7. The majority (*n* = 772, 95.5%) believed that HIV-positive learners should be allowed to attend school with HIV-negative learners, 771 (96.4%) indicated that they should be allowed to continue studying after testing HIV-positive, 734 (91.5%) said that HIV-positive learners could share a class with their children at school, and 746 (93.0%) said that they could be friends with their children.

However, the parents had less favorable attitudes on issues such as their children dating an HIV-positive learner; almost half (*n* = 378, 47.3%) did not approve of an HIV-positive learner having a romantic relationship with their children. Less than half (*n* = 355, 44.4%) had less favorable attitudes towards disclosure of HIV status (Table 6).

## 4. Discussion

Parents of adolescent children attending high schools in rural and urban districts in South Africa participated in a survey to assess the acceptability of the proposed provision of HTC at school to target high school learners. We found that although over three-quarters (78.9%) were biological parents, the majority (68.3%) were biological mothers. With regard to the family structure, we found that an equal proportion (41%) of households consisted of father and mother and mother alone, respectively, while 13.9% consisted of grandmothers, aunts, uncles, and older siblings. This suggests that the father is only present in 40% of the households. The family composition in the current study has implications for parent-child communication, since there is evidence that family structure is important in determining young people's sexual behavior [13]. The results further showed that over half of the parents were unemployed and the main sources of income were the old age and child support social grants. Parent-child interactions are also highly affected by poverty, and economic constraints make it difficult for parents to remain together and spend time with their children if fathers have to seek work elsewhere [13, 14]. In the current study, employed mothers were mostly likely to be employed as domestic workers who lived with the employers and return home on weekends or at the end of the month.

Open discussions between parents and children are crucial for the implementation of the proposed HTC in schools. We found that a high proportion (81.5%) of parents had discussed at least one sexual topic with children under their care at some time. This was despite the structure of the family not being ideal for positive parent-child communication [13]. As mentioned, two-thirds of the households did not have a father; nevertheless, the results showed that most of the discussions regarding sexuality were carried out by the biological mothers. This finding is in line with previous data which indicates that most parent-child communication about sexual and reproductive health issues is between the mothers and their adolescent children [15–17]. It should be noted that the proportion of parents who had communicated with their children at any time was high, although half indicated that it was not easy to discuss sexual topics with children. Therefore, there is a need for intervention programs to target parental knowledge, self-efficacy, and comfort to communicate with their children about sexuality [18]. The findings suggest that discussions on sexual topics take place when the parent deems them necessary; in the current study, over half of the parents initiated the discussions to prepare their children for the adolescent stage. Parents in a study conducted in Tanzania reported that they felt compelled to have the discussion on HIV to protect the child from HIV infection [19].

Various stakeholders, including learners, identified parental involvement as a key element for the successful implementation of HTC at school [1]. However, their attitudes and practices towards HTC are as equally important, as their personal practices might influence their support of HTC at school. We found high uptake of HIV testing among parents, with 87.9% reporting being tested for HIV at some time. Although only half of the parents were of the opinion that it was easy for adults to be tested for HIV, we found that the uptake of HIV testing was high. This indicates that adults perceived HIV testing among healthy adults as important. The high uptake of HIV testing has implications for the HTC campaigns in South Africa, with 84.3% of the parents indicating that they were tested in order to know their HIV status, and attests to the resounding success of the national HTC campaign. According to Simbayi and colleagues [20], South Africa has one of the highest proportions in the world of people who have been tested for HIV and know their status. Recent data from a study conducted in Gauteng province, South Africa, attributed the increased uptake of HTC to HIV campaigns that create awareness of HTC services in the communities [21]. However, the small proportion of parents who had never been tested is important as well, particularly when they showed low personal risk perception of HIV transmission. The perception of having no risk of HIV infection is a key reason for not being tested among different populations in South Africa [22, 23].

Madiba and Mokgatle [1] found that low personal risk perceptions of HIV infection were the main reasons for not being tested among learners. Although the current study did not attempt to link parents to their own children, as it was a follow-up study of a previous survey on learner's acceptability of HTC, we did plan to compare the two sets of results, because the success of the proposed provision of HTC would likely be influenced by both. We found that over 84% of the parents reported being tested for HIV at some time, while only half of the learners had been tested for HIV [1]. The high uptake of HIV testing has implications for the proposed provision of HTC at school, as it is likely that the positive attitudes and practices of parents towards HTC will influence their children to adopt HTC at school.

Our data also indicate a high level (92.9%) of acceptability of the provision of HTC services for learners in high schools among parents. The high level of acceptability of HTC at school and the high uptake of counseling and testing among parents suggest that parents view HIV testing as important not only for themselves but also for their children. We found that the level of acceptability of the proposed HTC among parents was much higher than that found among the learners (92.9% versus 76.9%). The current study did not investigate why parents support the provision of HTC at school; however, learners supported HTC at school because it was appropriate and convenient and was intended solely for them [1, 7]. The results further showed that over 87% of parents would be comfortable with their child receiving HTC at school, and this was slightly higher than the students' willingness to be tested at school (87% versus 71.8%) [1]. Obtaining parental consent for learners to test at school was one of the arguments for the provision of HTC at school. Over 40% of the parents felt that learners need parental consent for HTC at school. The belief that parents need to provide consent is in contrast to the South African Children's Act, which states that a child over 12 years may consent for an HIV test [24]. This finding has implications for the implementation of HTC at school and could be one of the major barriers to successful implementation. Govindasamy and colleagues [25] argue that the appropriate age at which learners can provide consent warrants further rigorous investigation.

The issuing of HTC results after testing at school is one of the key issues that might warrant further investigation because 39% of the parents felt that the results should be given to the child in the presence of the parent. We found that preference for issuing HTC result was independently associated with the need for parental consent at multivariate analysis. Parents who preferred that the HTC results should be given to the child with the parent present were four times more likely to suggest parental consent for HTC than those who preferred that HTC results should be given to the child alone. The survey conducted with the learners reported similar findings, with the majority of the learners indicating that they would prefer to receive their HTC results in the presence of their parents [1]. Although there is a level of agreement between parents and learners on the involvement of parents in the issuing of HIV test results, this might also be viewed as a potential barrier to the provision of HTC at school in view of the fact that a learner above 12 years can consent to a test for HIV. Therefore, the issuing of HTC test results is an important consideration for the success of HTC at school, and not only the views of both the parents and learners but also the Children's ACT's pronouncement should inform the implementation of HTC at school. The role of the parent in this regard will have to be clearly defined, and stakeholders should be engaged to start conversations around the Children's ACT in order to educate parents, children, and the community at large.

One of the arguments regarding the appropriateness of HTC at school among key stakeholders was that children who test positive would be stigmatized. Learners shared this concern and were anxious that other students might witness their reaction to positive test results and subject them to stigma and discrimination [1, 7]. Given the important role ascribed to parents for the roll-out of HTC, it was also argued that parental negative attitudes towards learners who test positive would influence acceptability of HTC at schools negatively. The results showed that the majority of parents believed that HIV-positive learners should be allowed to attend school with HIV-negative learners, share a class with their children at school, and be friends with their children. However, the parents had less favorable attitudes towards the issue of their children dating an HIV-positive learner, with almost half (47.3%) claiming they would not approve of a romantic relationship between their children and an HIV-positive learner. This finding supports the argument that the provision of HTC could subject children living with HIV to stigma and discrimination. Therefore, in preparing for the roll-out of the HTC at school, the program should also focus on stigma reduction interventions to create awareness and tolerance and empower parents and learners to accept living with HIV [1]. In order to protect learners from stigma and discrimination, parents did not support disclosure of HIV status to other children and teachers after testing HIV-positive at school.

### 4.1. Study Limitations

A key limitation of the study is information bias; because the questionnaire was self-administered at home, participants might have misunderstood some of the questions. We found that parents often misunderstood the question asking them about their age; instead of their own age, they provided the age of the child who delivered the questionnaire, hence the missing values for the age variable. We acknowledge that this might be the reason why age is not normally distributed in the sample. The study might also suffer from recall bias, social desirability recall, and self-report bias. However, some measures were taken to reduce recall by asking few questions that required recall. One other limitation of the study was a low response rate; 1200 questionnaires were distributed but only 805 were returned, with a response rate of 67%, but the investigators had no control over the delivering and completion of the questionnaire. The language (English) of the questionnaire might have influenced the low response rate as parents who are not well literate might have decided not to respond. The study design was cross-sectional and quantitative; as a result, the reasons for high acceptability of HTC at school could not be established. There is a need for additional mixed method studies to further explore this topic, especially because the sample size is small and cannot be generalized to all parents in Gauteng and North West provinces.

### 4.2. Conclusion

The vast majority of parents were in support of the proposed implementation of HTC in high schools. The findings revealed that parents felt that they had a role to play in the implementation of HTC. They wanted to be involved by providing consent for their children to be tested at school, to be there when their child receives their HIV test results, and to support a child who tested positive for HIV. However, these views did not apply to all parents, as some felt that the child does not need parental consent to be tested at school and that the HIV test results should be provided to the child alone at school. Parents had not received any information and had not been consulted concerning the proposed roll-out of HTC in high schools, but the draft DBE Policy on HIV, STIs, and TB states that parents would be trained and guided to participate in the school response to HIV and play a supporting role to children.

Open discussion between parents and children is a crucial consideration for the successful implementation of the proposed HTC in high schools. Our findings suggest that parent-child discussions on sexual topics take place when the parent deems them to be necessary; for example, they wanted to discuss the HIV test results with their children after being tested at school as a way of supporting the child, particularly after a positive test result. The findings of this study can inform the development of appropriate interventions that empower parents with adequate skills to initiate or improve discussions about HIV testing and other sexual matters with children, and this will be a key feature of their supportive role in the implementation of HTC in high schools. There should be provisions from the Departments of Health and Basic Education to create awareness and educational interventions for parents.

## Figures and Tables

**Table 1:** Sociodemographic characteristics of participants.

	Frequency	Percent
*Gender (n = 801)*		
Female	691	86.3
Male	110	13.7
*Age category (n = 734)*		
20–35 years	136	18.5
36–45 years	300	40.9
46–55 years	210	28.6
56–65 years	60	8.2
66–89 years	28	3.8
*Marital status (n = 803)*		
Married	327	40.7
Single	359	44.7
Widowed	71	8.9
Divorced	46	5.7
*Education (n = 804)*		
No schooling	28	3.5
Primary school	42	5.2
Secondary school	324	40.3
Completed grade 12	301	37.4
Tertiary	109	13.6
*Relationship to learner (n = 802)*		
Mother	548	68.3
Father	85	10.6
Grandparent	55	6.9
Aunt	53	6.6
Uncle	9	1.1
Brother	12	1.5
Sister	35	4.4
Guardian	5	0.6
*Family structure (n = 801)*		
Mother alone	331	41.3
Father alone	25	3.1
Mother and father	334	41.7
Grandparents	71	8.8
Other relatives	26	3.3
Older sibling	14	1.8
*Employment status (n = 805)*		
Unemployed	439	54.5
Employed	366	45.5
*Monthly income of employed parent (n = 337)*		
Less than R1000	30	8.9
R1100–2000	62	18.4
R2100–3000	89	26.4
R3100–5000	96	28.5
More than R5000	60	17.8
*Source of income of the unemployed parent (n = 406)*		
Old age grant	70	17.2
Child support grant	299	73.7
Child support and old age grant	6	1.5
Other	31	7.6

**Table 2:** Parents' response on discussions about sexuality with children

	Frequency	Percent
*Talk to child about sexuality (n = 805)*		
No	153	19.0
Yes	652	81.0
*Easy to discuss sexuality related topics (n = 804)*		
No	396	49.3
Yes	408	50.7
*Triggers of the discussion on sexuality (n = 801)*		
The child had reached adolescent stage	293	36.6
I thought the child was sexually active	68	8.5
To prepare the child for adolescent stage	440	54.9

**Table 3:** The HIV testing practices of parents.

	Frequency	Percent
*Ever tested for HIV (n = 800)*		
No	97	12.1
Yes	703	87.9
*Easy for adults to test (n = 798)*		
Not easy at all	367	46.0
Easy	282	35.3
Not sure	149	18.7
*HIV testing history (n = 702)*		
Three months ago	243	34.6
Six months ago	131	18.7
One year ago	195	27.8
More than two years ago	133	19.0
*Location where last tested for HIV (n = 704)*		
Hospital	101	14.4
Clinic	420	59.7
Private practitioner	82	11.7
HIV testing campaign	93	13.2
Work wellness	8	1.14
*Reason for having tested in the past (n = 701)*		
Wanted to know status	594	84.3
Was sick and referred by a doctor	29	4.1
Was pregnant	75	10.6
Applying for an insurance	3	0.4
Partner was sick	4	0.6
*Reason for not having tested for HIV in the past (n = 96)*		
I am scared to test	20	20.8
I trust my partner	12	12.5
Never think about HIV testing	42	43.8
I don't believe I can have HIV	22	22.9

**Table 4:** Parent's views about the provision of HTC for learners at school.

	Frequency	Percent
*Easy for learners to test for HIV (n = 795)*		
Not easy at all	389	48.9
Easy	230	28.9
Not sure	176	22.2
*HTC at school is a good idea (n = 799)*		
No	38	4.7
Yes	742	92.9
Not sure	19	2.4
*It is okay for your child to test at school (n = 799)*		
No	50	6.3
Yes	701	87.7
Not sure	48	6.0
*Learners need parental consent to test (n = 794)*		
No	365	46.0
Yes	322	40.6
Not sure	107	13.4
*Preference for issuing of HIV test results (n = 797)*		
Child alone at school	375	47.0
Child with parent	314	39.4
Post	108	13.6
*Learners should disclose to parents (n = 798)*		
No	253	31.7
Yes	545	68.3
*Learners should disclose to teachers (n = 796)*		
No	402	50.5
Yes	266	33.4
Not sure	128	16.1
*Learners should disclose to other learners (n = 799)*		
No	481	60.2
Yes	210	26.3
Not sure	108	13.5

**Table 5:** Multivariate logistic regression of factors associated with need for parental consent.

Need for parental consent	Odds ratio	Std. err.	*z*	*P* > *z*	[95% conf. interval]
Preference for HIV test results	1.76	0.186	5.38	0.000	1.43–2.17
Relationship to learner	0.92	0.049	−1.44	0.149	0.83–1.02
Family setup	0.95	0.054	−0.73	0.468	0.85–1.076

**Table 6:** Attitudes of parents towards the interaction of HIV-positive learners and their children at school.

	Agree	Disagree	Unsure
HIV-positive learners should attend school	772 (95.5)	8 (0.99)	25 (3.1)
It is okay that an HIV-positive learner should share a class with my child	734 (91.5)	29 (3.6)	39 (4.9)
HIV-positive learners should be allowed to keep their status secret	555 (69.3)	147 (18.4)	99 (12.4)
If a learner is HIV-positive, she/he should be allowed to continue studying	771 (96.4)	5 (0.6)	24 (3.0)
It is okay that an HIV-positive learner can be a friend to my child	746 (93.0)	16 (2.0)	40 (5.0)
My child can have a boyfriend/girlfriend who is an HIV-positive learner	166 (20.8)	378 (47.3)	256 (32)
HIV positive people should disclosure their HIV status	254 (31.8)	355 (44.4)	190 (23.8)
